# HLA-II-Dependent Neuroimmune Changes in Group A Streptococcal Necrotizing Fasciitis

**DOI:** 10.3390/pathogens12081000

**Published:** 2023-07-31

**Authors:** Ganesh Ambigapathy, Santhosh Mukundan, Kumi Nagamoto-Combs, Colin K. Combs, Suba Nookala

**Affiliations:** Department of Biomedical Sciences, University of North Dakota, Grand Forks, ND 58202, USA; ganeshneuro@gmail.com (G.A.); santhosh.mukundan@und.edu (S.M.); kumi.combs@und.edu (K.N.-C.); colin.combs@und.edu (C.K.C.)

**Keywords:** Group A Streptococcus, HLA-II, gliosis

## Abstract

*Streptococcus pyogenes* (Group A Streptococcus, GAS) bacteria cause a spectrum of human diseases ranging from self-limiting pharyngitis and mild, uncomplicated skin infections (impetigo, erysipelas, and cellulitis) to highly morbid and rapidly invasive, life-threatening infections such as streptococcal toxic shock syndrome and necrotizing fasciitis (NF). HLA class II allelic polymorphisms are linked with differential outcomes and severity of GAS infections. The dysregulated immune response and peripheral cytokine storm elicited due to invasive GAS infections increase the risk for toxic shock and multiple organ failure in genetically susceptible individuals. We hypothesized that, while the host immune mediators regulate the immune responses against peripheral GAS infections, these interactions may simultaneously trigger neuropathology and, in some cases, induce persistent alterations in the glial phenotypes. Here, we studied the consequences of peripheral GAS skin infection on the brain in an HLA-II transgenic mouse model of GAS NF with and without treatment with an antibiotic, clindamycin (CLN). Mice expressing the human HLA-II DR3 (DR3) or the HLA-II DR4 (DR4) allele were divided into three groups: (i) uninfected controls, (ii) subcutaneously infected with a clinical GAS strain isolated from a patient with GAS NF, and (iii) GAS-infected with CLN treatment (10 mg/kg/5 days, intraperitoneal). The groups were monitored for 15 days post-infection. Skin GAS burden and lesion area, splenic and hippocampal mRNA levels of inflammatory markers, and immunohistochemical changes in hippocampal GFAP and Iba-1 immunoreactivity were assessed. Skin GAS burden and hippocampal mRNA levels of the inflammatory markers S100A8/A9, IL-1β, IL-33, inflammasome-related caspase-1 (Casp1), and NLRP6 were elevated in infected DR3 but not DR4 mice. The levels of these markers were significantly reduced following CLN treatment in DR3 mice. Although GAS was not detectable in the brain, astrocyte (GFAP) and microglia (Iba-1) activation were evident from increased GFAP and Iba-1 mRNA levels in DR3 and DR4 mice. However, CLN treatment significantly reduced GFAP mRNA levels in DR3 mice, not DR4 mice. Our data suggest a skin–brain axis during GAS NF, demonstrating that peripherally induced pathological conditions regulate neuroimmune changes and gliotic events in the brain.

## 1. Introduction

*Streptococcus pyogenes*, also known as Group A Streptococcus (GAS), is a Gram-positive β-hemolytic bacterium responsible for a spectrum of infections ranging from self-limiting pharyngitis and mild, uncomplicated skin infections (impetigo, erysipelas, and cellulitis) to highly morbid and rapidly invasive life-threatening infections such as streptococcal toxic shock syndrome (STSS) and necrotizing fasciitis (NF), a dominant subset of necrotizing soft tissue infections (NSTI) [1]. In most cases, prompt intervention strategies, including surgical debridement, antibiotics (combination of clindamycin and penicillin), and management by hyperbaric oxygen therapy, lead to effective resolution of the infection and improved outcomes. However, antibiotic-resistant GAS strains are emerging, causing prolonged or repeated GAS infections and presenting a potential threat to public health [2,3]. Furthermore, the intracellular lifestyle of GAS represents hard-to-reach niches and complicates antibiotic access, leading to treatment failure and recurrent episodes induced by endogenous reservoirs [4]. GAS mimics of host proteins emerging from recurrent infections are not uncommon and can prime individuals for short- and long-term post-streptococcal autoimmune sequelae that include arthritis, glomerulonephritis, guttate psoriasis, acute rheumatic heart fever, and a temporal association with Sydenham’s chorea, with potential prolonged complications including rheumatic heart disease and clinically heterogeneous pediatric autoimmune neuropsychiatric disorders associated with streptococcal infections (PANDAS) [5,6,7,8,9,10]. A significant risk for seizures has also been reported to be associated with GAS infections [11]. Several host genetic factors affect GAS NF/STSS pathogenesis. Specifically, polymorphisms in host HLA-II molecules directly determine the risk and significantly influence the outcomes and severity of GAS NF/STSS and post-streptococcal sequelae [12]. 

It has been established that peripheral inflammation induced by bacteria/viruses or their products contributes to neuroinflammation, neurodegeneration, and related cognitive dysfunction. For instance, systemic exposure to the Gram-negative bacterial endotoxin LPS has been widely used to demonstrate neuroinflammation and associated behavioral symptoms, including sickness behavior [13]. GAS is not distinctively neurotropic, yet recurrent GAS infections are frequently associated with several neurological dysfunctions such as Tourette’s syndrome, a PANDAS subgroup of tic disorders, attention disorders [14,15,16,17], meningitis [18], and seizure risks in some cases [11]. Intriguingly, while post-streptococcal neurological and neuropsychiatric conditions are associated with an autoimmune response due to cross-reactivity between host and GAS proteins (molecular mimicry) [19,20], it is not known whether invasive GAS NF can induce concomitant neuroinflammatory changes and cause long-lasting brain changes that result in behavioral and cognitive dysfunction. Furthermore, there is a lack of information regarding the neuroprotective potential of Clindamycin (CLN), well-established as an effective antibiotic for treating invasive GAS infections [21]. 

The use of HLA-II transgenic mice as a novel in vivo disease model has been reported in various studies [22], including those examining regulation of autoimmunity [23], experimental autoimmune encephalomyelitis [24], the effect of T-cell epitope-based HIV vaccines [25], immune responses against Aβ1-42 [26], the pathogenesis of multiple sclerosis [27], and streptococcal infections [28]. Specifically, HLA-II mice expressing the DR3 allele have been evaluated in behavioral and immune responses to the whey allergen β-lactoglobulin [29], experimental autoimmune encephalomyelitis and high fat-induced multiple sclerosis susceptibility [24,30], lupus [31], and a staphylococcal superantigen-induced skin wound infection model [32]. Increased DR3 (DRB1*0301) allele frequencies have been reported in rheumatic heart disease, an autoimmune post-streptococcal sequela [33]. Previous epidemiological studies from our laboratory demonstrated an interaction between NF and severe streptococcal diseases, and individuals with DR3/DQ2 (DRB1*03/DQB1*0201) haplotype were much less likely to suffer from NF in the absence of multiple organ failure [12]. Humanized HLA-II DR4-expressing mice have been reported as preclinical models in various studies, including rheumatoid arthritis [34,35,36], *Chlamydia trachomatis* infections [37], and vaccine responses against pulmonary Coccidioides infection [38,39]. Norrby-Teglund et al. reported that DR4 alleles preferentially presented the GAS superantigen SpeC compared to SpeA, suggesting that allelic polymorphisms influence GAS superantigen-induced responses [40]. The genetic susceptibility to the post-streptococcal sequela rheumatic fever (in the absence of rheumatic heart disease) has been linked to individuals with the haplotype DR4/DQ4 (DRB1*04/DQA1*0401), while the haplotype DR3/DR4 (DRB1*0301/DRB1*0401-2) was frequently associated with combined rheumatic fever and Sydenham’s chorea [41,42]. Based on the substantial evidence that HLA-II allelic variations influence peripheral immune responses to invasive GAS infections [12] and HLA-II expressing cells reshape T-cell immune responses in the brain during peripheral insults [30], we hypothesized that systemic GAS infections mediate neuroinflammatory changes that could be HLA-II dependent. Utilizing humanized HLA-II DR3 and DR4 transgenic mouse models of GAS NF/STSS, here we tested whether skin GAS infection induces neuroimmune changes that could be attenuated by CLN treatment. 

## 2. Materials and Methods

### 2.1. Ethics Statement

All the animal experiments described in the current study were conducted per the recommendations in the *Guide for the Care and Use of Laboratory Animals* of the National Institutes of Health. The breeding and maintenance of mice and all experimental protocols were approved by the University of North Dakota Institutional Animal Care and Use Committee, protocols 1608-7C and 1704-3. 

### 2.2. In Vivo GAS Infections

Male and female mice expressing HLA-II DRB1*0301 (DR3) or DRB1*0401 alleles (DR4) were used. DR3 and DR4 mice were originally generated in the laboratories of Drs. David Bradley at the University of North Dakota and C.S. David, Mayo Clinic, Rochester, MN [27,43]. Surface expression of HLA-II DR was confirmed by flow cytometry using an LSR-II flow cytometer (BD Biosciences, Franklin Lakes, NJ, USA) after staining whole blood with allophycocyanin-labeled anti-HLA-DR antibody (Clone L243) (eBioscience-Thermo Fisher, Waltham, MA, USA, or Tonbo Biosciences (Cytek Biosciences, San Diego, CA, USA).

A clinical GAS strain (M1 GAS 2006) originally isolated from an NSTI patient (INFECT Consortium) [44,45] was used for in vivo studies in the HLA-II transgenic mice. Bacteria growth, preparation of mice, and infections were performed as described previously [46,47,48]. Briefly, GAS 2006 was cultured under static conditions at 37 °C for 17 h in THY medium (BD Bacto Todd-Hewitt broth (Cat# 249240) containing 1.5% (*w*/*v*) Bacto yeast extract (Cat# 212750), Fisher Scientific, Waltham, MA, USA)). The bacteria were centrifuged for 10 min at 1800 rpm (610× *g*), washed three times, and re-suspended in sterile, endotoxin-free Dulbecco’s phosphate-buffered saline (DPBS) (Fisher Scientific). GAS bacteria were diluted to the desired optical density at 600 nm (OD_600_ adjusted to yield ~1–5 × 10^8^ CFU/0.1 mL). Actual inocula were determined by plating serial dilutions on sheep blood agar plates (Fisher Scientific). Age- and sex-matched 20–24-week-old, HLA-II DR3 or DR4 mice (*n* = 3–6 mice per group) were used. Because CLN is strongly recommended as the first line of treatment for NSTI [21], we chose CLN (Gold Biotechnology, Olivette, MO, USA) to treat GAS infections in our mouse models of GAS NF. Seventy-two hours post-infection, mice within each strain were randomly assigned to receive either treatment with CLN administered intraperitoneally (IP) at 10 mg/kg body weight (in 100 µL) daily for five days or DPBS. Uninfected control mice also received DPBS. Mice were monitored twice daily for 15 days for survival, and the skin lesion area was measured using digital calipers [46,47]. 

### 2.3. Tissue Collection

At the end of the experiment, mice were euthanized by CO_2_ inhalation, and blood was drawn through cardiac puncture for bacteremia estimations. The spleen, brain, and necrotic skin were recovered from each mouse under aseptic conditions. The necrotic skin was homogenized using a motorized homogenizer (Omni International, Marietta, GA, USA), and the GAS burden was enumerated by preparing tenfold dilutions in DPBS and plated on sheep blood agar as described previously [47,48]. The brains were hemisected, and the hippocampus was isolated from the right hemisphere. The right hippocampus and the spleen were stored in RNAlater (Invitrogen, Thermo Fisher) for gene expression studies. The left hemispheres were fixed in 4% paraformaldehyde (pH 7.4) for histological analyses.

### 2.4. Gene Expression Changes in the Spleen and Hippocampus by Quantitative Real-Time PCR 

Total RNA from the spleen and hippocampal samples were isolated using an RNeasy Mini Kit (Qiagen, Germantown, MD, USA) following the manufacturer’s protocols. RNA concentrations were analyzed using a Nanodrop spectrophotometer (ND-1000, Thermo Fisher). A total of 0.2–1 µg of RNA was pre-treated with DNase and used for cDNA synthesis using the iScript cDNA Synthesis Kit (Bio-Rad Laboratories, Irvine, CA, USA). Quantitative real-time PCR was performed on a Bio-Rad CFX 384 Real-Time PCR System using iTaq-SYBR Green Supermix (Bio-Rad) with specific primer sets (Table 1). Relative gene expression was calculated using the comparative 2^−ΔΔCq^ method [49]. Data were normalized against a set of 4 reference genes: ribosomal protein L0 (RPL0), ribosomal protein L27 (RPL27), glyceraldehyde 3-phosphate dehydrogenase (GAPDH), and beta-actin (Actb). The fold change was calculated relative to the average of the uninfected controls, and the results are presented as a relative expression. Primer details are provided in Table 1. 

### 2.5. Histological Tissue Preparation and Immunostaining

Paraformaldehyde-fixed brains from uninfected or GAS-infected mice with or without CLN treatment were prepared for immunohistochemical staining as described previously [50]. Briefly, tissues were equilibrated in 30% sucrose prepared in PBS and embedded in a 15% gelatin (in 0.1 M phosphate buffer, pH 7.4) matrix to form a sample block for the simultaneous handling of multiple brain samples. The samples were arranged in such a way as to facilitate the comparison of different conditions on a single gelatin section. The block was immersed in a 4% paraformaldehyde solution for 3–4 days to fix the gelatin matrix and equilibrated in 30% sucrose changed every 3–4 days for two weeks. The block was completely immersed in the sucrose solution. The cryoprotected blocks were then flash-frozen using dry ice and sectioned at 40 μm using a Leica SM2000R sliding microtome (Leica Biosystems, Deer Park, IL, USA). For immunohistochemistry, sections were first incubated in 0.3% H_2_O_2_ in PBS for 10 min at room temperature to quench endogenous peroxidase activity. Subsequent rinsing, blocking, and antibody incubation were performed with PBS containing 5% normal goat serum, 0.5% BSA, and 0.1% TritonX-100. Anti-ionized calcium-binding adaptor molecule 1 (Iba-1, FUJIFILM Wako Chemicals USA, Richmond, VA, USA) and anti-glial fibrillary acidic protein (GFAP, Cell Signaling Technology, Danvers, MA, USA) antibodies were diluted to 1:1000 and used to incubate tissue sections overnight at 4 °C. A Vectastain ABC Elite Kit (Vector Laboratories, Newark, CA, USA) was used to visualize the immunoreactivity with diaminobenzidine (DAB) as the chromogen according to the manufacturer’s instructions. The sections were mounted onto gelatin-subbed glass slides, cleared in Histo-Clear (National Diagnostics, Atlanta, GA, USA), and cover-slipped using VectaMount (Vector Laboratories).

### 2.6. Quantification of GFAP and Iba-1 Immunoreactivity

Immunohistochemically stained slides were digitalized on a Hamamatsu NanoZoomer 2.0-HT slide scanner (Hamamatsu Photonics, Hamamatsu City, Japan) at 40× magnification. The quantification of GFAP and Iba-1 positive cells was performed on whole-slide images (n = 3–6 mice/group, 2–3 serial sections/mouse) using the open-source digitalized image analysis platform QuPath (v.0.3.2) [51]. For quantification, the image type was set to brightfield H-DAB. To calibrate the intensity of DAB staining and reduce background noise, areas with distinct positive and negative DAB staining were selected, images were preprocessed, and RGB values for the DAB stain were separated into their respective components by applying QuPath’s color deconvolution feature. Using annotation tools in QuPath, the hippocampus region was manually marked as the region of interest for analysis. A thorough manual inspection was performed to exclude any sample that did not exhibit regular boundaries. To consistently capture positive signals that extended beyond the cell body and into the processes (in the case of GFAP), we chose superpixel-based (simple linear iterative clustering, SLIC) segmentation for quantification, as previously described [52,53,54]. QuPath clusters similar pixels into superpixels based on the RGB values initially set for the DAB stain. We elected to use 25 mm^2^ for the superpixel size to obtain a precise resolution of the positively stained pixels. QuPath’s built-in intensity feature was then applied to the segmented superpixels to classify them as either “positive” or “negative,” involving simple thresholding of a single measurement. Artifacts and blank spaces were selected and ignored from threshold settings and ensuing analysis. The threshold classification was manually checked to avoid false positives or negatives and ensure the settings captured all positively stained cells in the hippocampus annotated region. Data are presented as a positive percentage of anti-GFAP and anti-Iba-1-stained pixels [55].

### 2.7. Generation of Heatmaps and Correlation Plots

Hierarchical clustering and heatmap visualization (Figure 6A,B) and similarity matrix correlation plots (Figure 6C,D) of the expression data from the spleen (Sp) and hippocampus (Hc) were generated using Morpheus (Broad Institute; https://software.broadinstitute.org/morpheus, accessed on 10 February 2022). The hierarchical clustering for heatmaps was made using the following parameters: one minus Spearman rank correlation as the metric, average for the linkage method, and clustering by rows and columns [29]. To uncover distinct correlations in the mRNA expression patterns within the data set and to enable visualization of the extent of correlation among the markers (ranging from -1 (blue, negative) to +1 (red, positive)), we employed similarity matrix tools (Spearman rank correlation as the metric and computed for rows) followed by hierarchical clustering.

### 2.8. Statistical Analysis

Values are presented as mean ± SEM and were analyzed using the unpaired Student’s *t*-test or one-way ANOVA with uncorrected Fisher’s LSD under a statistical threshold of *p* ≤ 0.05 using Prism^®^ 9.3.1 (GraphPad Software Inc. San Diego, CA, USA).

## 3. Results

### 3.1. CLN Attenuated Skin GAS Burden in HLA-II DR3 Mice

**Figure 1:**
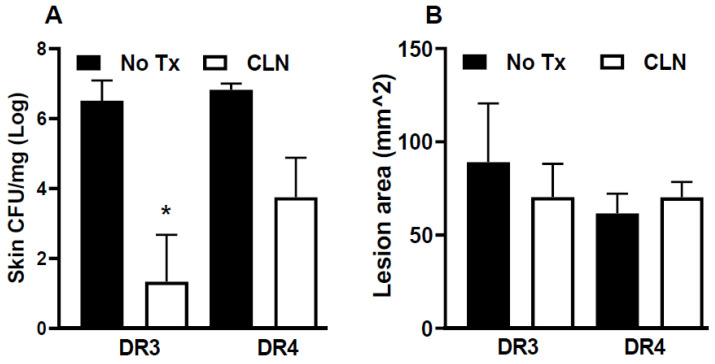
Skin CFU and lesion area in HLA-II DR3 and DR4 mice following subcutaneous GAS infection. HLA-II mice expressing DR3 or DR4 were infected subcutaneously with 1 × 10^8^ CFU of GAS 2006. Mice were either untreated (No Tx) or treated with clindamycin (CLN). At 15 days post-infection, lesions were excised to enumerate GAS burden (**A**) and lesion area (**B**). Data are presented as mean values ± SEM, n = 3–6, * *p* < 0.05, Student’s *t*-test.

The resolution of inflammation and tissue pathology mitigation depends on the infecting GAS strain and the host HLA-II context. Here, we investigated the therapeutic benefit of the standard CLN treatment in HLA-II DR3 or DR4 mice. At 15 days post-infection, GAS was not detectable in the blood or brain of untreated or CLN-treated mice. GAS persisted in the skin at the site of infection. The GAS burden was significantly reduced with CLN treatment in the HLA-II DR3 mice (*p* = 0.0174) but not in the DR4 mice (*p* = 0.0904), as shown in Figure 1A. Subcutaneous GAS infections led to the development of lesions at the site of infection that were not significantly altered following CLN treatment in DR3 or DR4 mice (Figure 1B).

### 3.2. CLN Treatment Differentially Altered mRNA Levels of Numerous Genes in Spleens from GAS-Infected HLA-II DR3 Mice

**Figure 2:**
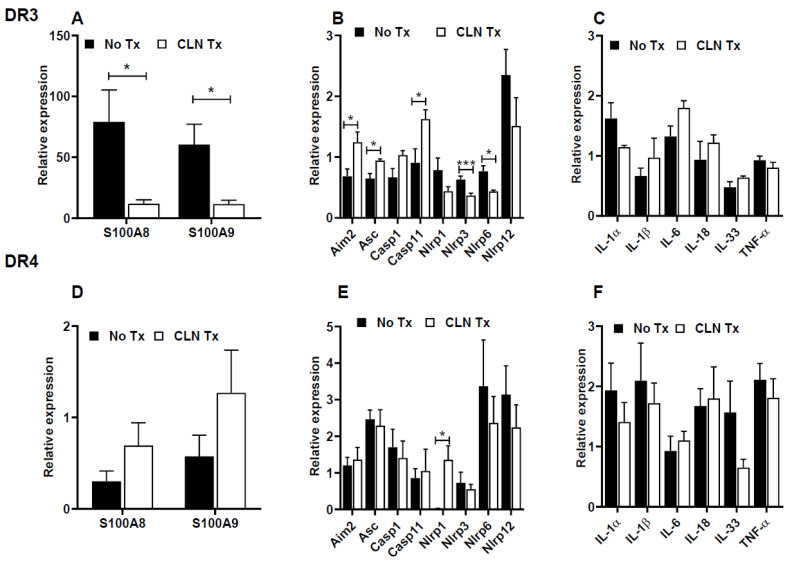
Evaluation of pro-inflammatory markers in the spleen following subcutaneous GAS infection and CLN treatment. The mRNA levels of S100A8 and S100A9 (**A**,**D**), inflammasome-related genes (**B**,**E**), and pro-inflammatory mediators (**C**,**F**) were determined by quantitative real-time PCR in the spleen from GAS-infected HLA-II DR3 (**A**–**C**) or DR4 (**D**–**F**) mice that were either untreated (No Tx) or treated with CLN. The fold change was calculated using the comparative 2^−ΔΔCq^ method after normalization against four housekeeping genes. Data are presented as mean values ± SEM, n = 4, * *p* < 0.05, *** *p* < 0.001, multiple unpaired *t*-test.

The prolonged skin GAS burden and unmitigated tissue pathology led us to investigate the changes in splenic mRNA levels of genes associated with inflammation, inflammasomes, and pro-inflammatory mediators. Specifically, we examined the changes in the mRNA levels of (a) the inflammatory markers S100A8 and S100A9; (b) the inflammasomes NLRP1, NLRP3, NLRP6, and NLRP12, and the inflammasome components inflammasome absent in melanoma (AIM2), apoptosis-associated speck-like protein containing a CAR domain (ASC), and caspase 1 (Casp1) and caspase 11 (Casp11); and (c) the pro-inflammatory mediators IL-1α, IL-1β, IL-6, IL-18, IL-33, and TNF-α. As shown in Figure 2A, GAS infections in DR3 mice induced a marked increase in the mRNA levels of S100A8 and S100A9 that were significantly reduced (S100A8, *p* = 0.0104, and S100A9, *p* = 0.0485) with CLN treatment. Elevated levels of S100A8 and S100A9, mainly derived from neutrophils and macrophages, have been implicated in inducing inflammasome activation and secretion of pro-inflammatory mediators [56]. Our data show that, consistent with the decrease in S100A8 and S100A9 mRNA levels in CLN-treated DR3 mice, mRNA levels of the inflammasomes NLRP1, NLRP3, NLRP6, and NLRP12 also decreased, with significant reductions in NLRP3 and NLRP6 (*p* = 0.009 and 0.011, respectively). Surprisingly, there were significant increases in the mRNA levels of the inflammasome component genes AIM2 (*p* = 0.0367), ASC (*p* = 0.0158), and Casp11 (*p* = 0.041) in CLN-treated DR3 mice (Figure 2B). Increases in Casp1 mRNA levels in CLN-treated DR3 mice compared to untreated mice were apparent. Yet, these differences did not reach statistical significance (Figure 2B). Intriguingly, CLN treatment did not significantly alter the mRNA levels of the inflammatory mediators IL-1α, IL-1β, IL-6, IL-18, IL-33, or TNF-α in the DR3 mice (Figure 2C). Despite comparable skin GAS burden and lesion area in GAS-infected DR3 and DR4 mice, GAS infections in DR4 mice did not elicit similar levels of splenic S100A8 or S100A9 mRNA expression (Figure 2D). It is noteworthy that the splenic S100A8 and S100A9 mRNA transcripts were severalfold less expressed in the DR4 mice compared to the DR3 mice, as indicated by the striking differences in the values represented on the Y-axis scale (Figure 2D). Furthermore, except for NLRP1, CLN treatment in DR4 mice did not significantly alter the mRNA expression of other inflammasome markers (NLRP1, *p* = 0.008, Figure 2E), or the pro-inflammatory mediators (Figure 2F).

### 3.3. CLN Treatment Differentially Altered mRNA Levels of Numerous Genes in Hippocampi from GAS-Infected HLA-II DR3 and DR4 Mice

**Figure 3:**
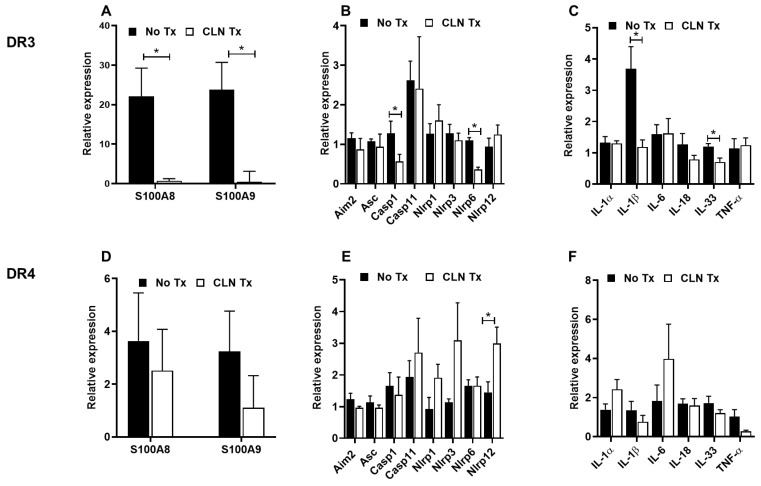
Distinct differences in hippocampal pro-inflammatory and inflammasome markers following subcutaneous GAS infection and CLN treatment. The mRNA levels of S100A8 and S100A9 (**A**,**D**), inflammasome-related genes (**B**,**E**), and pro-inflammatory mediators (**C**,**F**) were determined by quantitative real-time PCR in the hippocampus from GAS-infected HLA-II DR3 (**A**–**C**) or DR4 (**D**–**F**) mice that were either untreated (No Tx) or treated with CLN. The fold change was calculated using the comparative 2^−ΔΔCq^ method after normalization against four reference genes. Data are presented as mean values ± SEM, n = 4, * *p* < 0.05, multiple unpaired *t*-test.

To further examine the potential communication of the inflammatory responses to the brain, we assessed changes in the hippocampal mRNA levels of genes associated with inflammation, inflammasomes, and pro-inflammatory mediators, as described above. CLN treatment significantly reduced hippocampal S100A9 mRNA levels in the GAS-infected DR3 mice (*p* = 0.040, Figure 3A) and to a lesser extent the mRNA levels of S100A8 (*p* = 0.054, Figure 3A). However, except for NLRP6 (*p* = 0.002) and Casp1 (*p* = 0.034), CLN treatment did not significantly alter the mRNA expression of the hippocampal inflammasomes in GAS-infected DR3 mice (Figure 3B). Interestingly, mRNA expression levels of hippocampal IL-1β and IL-33 were significantly reduced in CLN-treated DR3 mice (*p* = 0.015, and *p* = 0.006 respectively, Figure 3C). There was a modest induction of S100A8 and S100A9 mRNA expression in GAS-infected DR4 mice; however, CLN treatment did not ameliorate these responses (Figure 3D). It is noteworthy that the hippocampal S100A8 and S100A9 mRNA transcripts were severalfold less expressed in the DR4 mice compared to the DR3 mice, as indicated by the striking differences in the values represented on the Y-axis scale (Figure 2D). The relative mRNA expression of NLRP1, NLRP3, NLRP12, and Casp11 showed an increase in CLN-treated DR4 mice; however, increases in NLRP12 levels alone were significant (*p* = 0.045, Figure 3E). CLN treatment in DR4 mice did not significantly alter the mRNA expression of the pro-inflammatory mediators (Figure 3F).

### 3.4. CLN Treatment Attenuated GFAP mRNA Levels in Hippocampi from GAS-Infected HLA-II DR3 Mice

**Figure 4:**
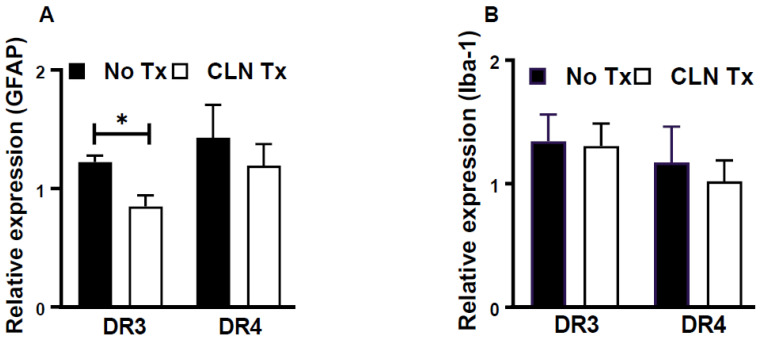
Glial activation as a marker of hippocampal inflammation following subcutaneous GAS infection and CLN treatment. GFAP (**A**) or Iba-1 (**B**) mRNA levels were determined by quantitative real-time PCR in the hippocampi from GAS-infected HLA-II DR3 or DR4 mice that were either untreated (No Tx) or treated with CLN. Bar graphs represent the fold change calculated using the comparative 2^−ΔΔCq^ method after normalization against four housekeeping genes and uninfected samples. Data are presented as mean values ± SEM, n = 4, * *p* < 0.05, unpaired *t*-test.

In the central nervous system, the microglia and the astrocytes play an essential role in brain/hippocampal innate immune responses. Therefore, hippocampal glial activation patterns following peripheral skin GAS infection were studied by quantifying mRNA levels of GFAP and Iba-1 for astrocytes and microglia, respectively, without or with CLN treatment. As shown in Figure 4A, CLN treatment significantly reduced mRNA levels of GFAP in DR3 mice (*p* = 0.013), while no such change was observed in DR4 mice (Figure 4A). Interestingly, Iba-1 mRNA levels were unaltered by CLN treatment in either the DR3 or DR4 mice (Figure 4B).

### 3.5. Increased GFAP Immunoreactivity in Hippocampi of GAS-Infected HLA-II DR3 Mice

**Figure 5:**
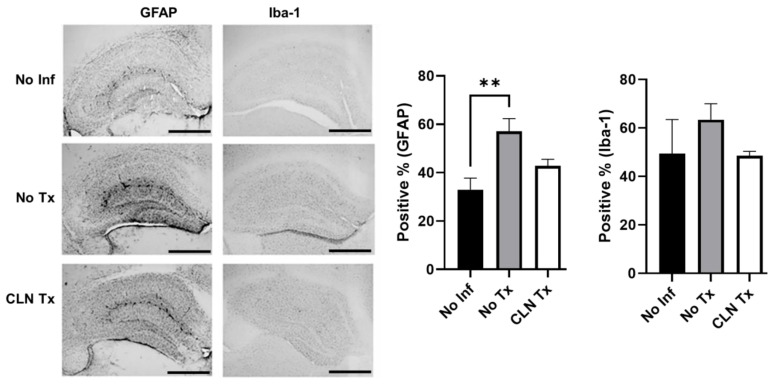
Increased GFAP immunoreactivity in hippocampi of GAS-infected HLA-II DR3 mice. Representative image (4× magnification, scale bar 500 μm) of immunohistochemical detection of the astrocyte marker GFAP and the microglial marker Iba-1 in the brain tissue from uninfected (No Inf) or GAS-infected −/+ CLN Tx HLA-II DR3 mice collected 15 days post-infection. GFAP and Iba-1 intensities were quantitated using QuPath. Bar graphs represent the positive percentage of stained pixels calculated using the QuPath software. Data are presented as mean values ± SEM, n = 3–4, ** *p* < 0.01, one-way ANOVA.

We next examined the immunohistochemical changes in GFAP and Iba-1 in DR3 mice to compare them to the mRNA changes. Immunohistochemistry demonstrated that skin GAS infection significantly increased hippocampal GFAP immunoreactivity (*p* = 0.002), which was not altered with CLN (*p* = 0.203, Figure 5). Consistent with the mRNA levels, Iba-1 immunoreactivity was unaltered in DR3 mice (Figure 5).

### 3.6. Analysis of mRNA Expression Patterns by Heatmap and Similarity Matrix Revealed Unique Clusters

**Figure 6:**
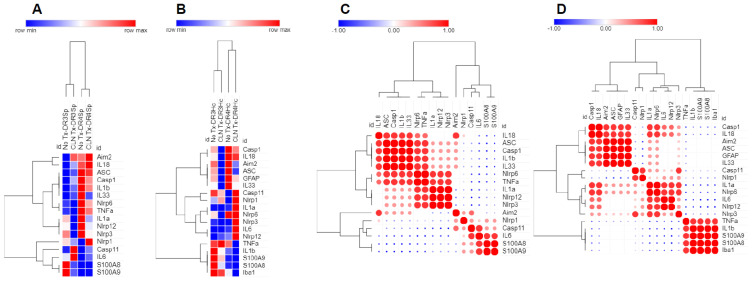
Generation of heatmaps and correlation plots. Hierarchical cluster heatmaps and correlation plots of the expression data from the spleen (**A**,**C**) and hippocampus (**B**,**D**) were generated using Morpheus (Broad Institute; https://software.broadinstitute.org/morpheus, accessed on 10 February 2022). The hierarchal cluster heatmaps were made using the following parameters: one minus Spearman rank correlation as the metric, average for the linkage method, and clustering by rows and columns. The color key displays the fold change values from minimum (blue) to maximum (red). The correlation heatmaps were created using the Morpheus similarity matrix tools, using the following parameters: Spearman rank as the metric and computed for the rows. The color key displays the relative color scheme of the Spearman rank correlation ranging from –1 (blue, negative) to +1 (red, positive).

To further discern the transcriptional regulation of splenic and hippocampal S100A8, S100A9, inflammasomes, and pro-inflammatory mediators in skin GAS infections, we performed a more in-depth analysis based on clusters or a similarity index of the mRNA expression profiles of the markers. The relative expression values in mRNA levels for the S100A8, S100A9, inflammasomes, and pro-inflammatory mediators were uploaded to the Morpheus software to generate heatmaps and a similarity matrix. As shown in Figure 6A, three main clusters were apparent in the heatmap of the spleen: cluster-1, made up of AIM2 and IL-18; cluster-2, made up of ASC, Casp1, IL-1β, IL-33, NLRP6, TNF-α, IL-1α, NLRP12, and NLRP3; and cluster-3, made up of NLRP1, Casp11, IL-6, S100A8, and S100A9. Clusters in the hippocampus included: cluster-1, made up of Casp1, IL-18, AIM2, ASC, GFAP, and IL-33; cluster-2, made up of Casp11, NLRP1, IL-1α, NLRP6, NLRP3, IL-6, and NLRP12; and cluster-3, made up of TNF-α, IL-1β, S100A9, S100A8, and Iba-1 (Figure 6B). The similarity correlation matrix identified which variables were positively or negatively related and to what extent, providing insights into underlying patterns within the data. It is notable from the Spearman rank correlation analysis that the expression of spleen S100A8 and S100A9 coordinated with IL-6, Casp11, and NLRP1 (Figure 6C), while hippocampal S100A8 and S100A9 coordinated with Iba-1 and the two most prominent pro-inflammatory mediators, TNF-α and IL-1β (Figure 6D).

## 4. Discussion

The severity of GAS infection outcomes depends on the heterogeneity of the GAS virulence factors and the host HLA-II allelic polymorphisms. GAS infections have been linked to a spectrum of neurological complications arising as direct effects of autoimmune reactions [20] and indirect effects from peripheral inflammation [57]. Dileepan et al. have demonstrated that intranasal GAS infections induce CNS complications, blood–brain barrier compromise, IgG deposition, microglial activation, and infiltration of a GAS-specific Th17 subset of CD4^+^ cells, despite the lack of viable GAS persistence in the brain tissue in C57BL/6, C57BL/6J, or SJL/J female mice [58]. To establish the relevance of our findings to human settings, we used humanized mice expressing HLA-II DR3 or DR4 as preclinical subcutaneous infection models of GAS NSTI with no apparent lethal systemic toxicity [12,28,47]. We assessed the effectiveness of CLN, a protein synthesis inhibitor antibiotic with a broad spectrum of activities, including activity against stationary growth-phase GAS and the suppression of virulent GAS toxins [59], that has been demonstrated in Swiss Webster and C57BL/6 mouse models of GAS myositis and subcutaneous infections [2,60,61]. In parallel with the findings reported by Andreoni et al. [2], our results show that CLN treatment does not eliminate skin GAS burden but likely attenuates the activity of GAS virulence factors.

It is well-established that the calgranulin S100A8/A9 complex is one of the biomarkers in sepsis and exerts its inflammatory role through TLR4 activation [62]. Consistent with the preferential reduction in the skin GAS burden in CLN-treated DR3 mice, elevated splenic and hippocampal S100A8 and S100A9 mRNA levels were significantly reduced in DR3 but not DR4 mice. These data emphasize HLA-II-dependent variations in the induction of S100A8 and S100A9 responses during skin GAS infections. The reasons underlying the differential induction of S100A8 and S100A9 responses in GAS-infected DR3 and DR4 mice are unclear and need further investigation. It will be important to assess the changes in the virulence capability of GAS bacteria in different hosts that might influence inflammation and outcomes. The disparity in CLN efficacy is also concerning and adds to the overriding effects of HLA-II allelic polymorphisms in shaping not just peripheral but also brain inflammatory responses during skin GAS infections.

In the present study, hippocampal mRNA levels of Casp11 and IL-1β were highly induced in GAS-infected DR3 mice, suggesting that despite the lack of viable GAS burden in the brain, GAS products or genetic material likely triggered these responses. The functional role of the AIM2 inflammasome and caspases in regulating astrogliosis has been reported [63,64]. In support of this notion, it is interesting to note from our similarity matrix that changes in the relative expression of GFAP showed positive coordination with the AIM2 inflammasome and associated partners ASC, Casp1, IL-33, and IL-18. Ma et al. demonstrated that the AIM2 inflammasome negatively regulates microglial activation in mouse models of experimental autoimmune encephalomyelitis [65]. The involvement of AIM2 in regulating microglia responses in GAS NF is unclear. In our study, skin GAS infection-induced mRNA expression of AIM2, ASC, and Iba-1 in DR3 and DR4 mice and their levels were not ameliorated with CLN in DR3 or DR4 mice. Monocytes and macrophages are the primary sources of the pro-inflammatory mediators IL-1β, IL-18, TNF-α, and IL-6, which are also central to GAS pathogenesis. TNF-α is mainly released by activated microglia, and it is well-known that the innate immune mechanisms and inflammasome signaling are mediated by microglia in the brain. Our data show hippocampal TNF-α, IL-6, and Iba-1 mRNA levels persisted despite CLN treatment in DR3 mice. In contrast, hippocampal mRNA levels of both TNF-α and Iba-1 showed modest decreases in CLN-treated DR4 mice. Increased hippocampal NLRP1 and NLRP12 mRNA expression was an unpredicted outcome of CLN treatment in GAS-infected DR3 and DR4 mice. It has been shown that the NLRP1 inflammasome is expressed by pyramidal neurons and oligodendrocytes in the brain [66], and increased NLRP1 expression has been reported in aging-related neuronal damage [67]. Among the inflammasomes, the cytosolic pathogen sensor NLRP12 has been implicated in maintaining gut homeostasis. It is different from other members of the NLR family due to its dual role in the activation and dampening of NF-κB signaling [68]. Sun et al. demonstrated that CLN treatment depleted secondary bile acid-producing gut bacteria and exacerbated campylobacteriosis in mice [69]. Furthermore, CLN treatment has been associated with an increased risk for pseudomembranous colitis, often caused by overgrowth of *Clostridium difficile* [70]. An open critical question is whether increased hippocampal NLRP1 and NLRP12 expression is an unintended consequence of CLN therapy by inducing gut microbiome changes and triggering gut dysbiosis, thereby exerting a potent impact on the simultaneous modulation of brain inflammasomes via the intricate bidirectional gut–brain axis. These assertions are ambitious and require further validation to establish their veracity. Further studies are also needed to elucidate the direct involvement of hippocampal NLRP1 and NLRP12 in regulating glial and neuronal responses in GAS infections.

We acknowledge the limitations of our study, including the small sample size and our mRNA-focused approach. Information about protein levels could have validated the mRNA findings and provided insights into protein abundance and functional outcomes. However, this proof-of-concept study demonstrates HLA-II-dependent neuroinflammation despite CLN therapy and might impactfully translate to other disease entities. In addition to HLA-II allelic variations, neuroinflammatory sequelae may be influenced by GAS strain variability. Therefore, in addition to incorporating protein-level analysis, such as Western blotting and ELISA, to assess the levels of inflammasomes and pro-inflammatory mediators (zymogen and mature forms), one future direction is to evaluate the neuroinflammation potential of several clinical GAS isolates across a battery of HLA-II transgenic mice. We considered HLA-II transgenic mice as a clinically relevant and translational model to study the neuroinflammation effects of peripheral inflammation involved in GAS infections because these mice mimic responses seen in humans [47,71]. Notwithstanding that the magnitude of responses to invasive skin GAS infections is directly linked to HLA-II allelic variations, it is understandable that a single gene cannot hold the key to all the intricacies of disease pathogenesis underlying GAS infections. Several genes related to an immune function whose loci are located within the central major histocompatibility complex region or in linkage disequilibrium with HLA-II genes might either counteract or exacerbate the overriding effects of HLA-II genes, thereby polarizing the immune responses and influencing outcomes [19].

In conclusion, our findings indicate that subcutaneous GAS infections that display systemic inflammation trigger the production of pro-inflammatory mediators and glial changes, despite the absence of viable GAS burden in the brain, raising the possibility of increased risk of neurological changes after invasive GAS infections. Future studies will determine the link between pro-inflammatory insults and long-lasting brain pathological changes with behavioral complications and cognitive dysfunction.

## Figures and Tables

**Table 1:** Sequences of primers used for gene expression analysis by quantitative real-time PCR.

Gene	Primer Sequence (Forward) (5′-3′)	Primer Sequence (Reverse) (5′-3′)
S100A8	GAGAAGGCCTTGAGCAACCTCATTG	CCTTGTGGCTGTCTTTGTGAGATG
S100A9	GCAAGAAGATGGCCAACAAAGCAC	TCAAAGCTCAGCTGATTGTCCTGG
IL-1α	AAGACAAGCCTGTGTTGCTGAAGG	TCCCAGAAGAAAATGAGGTCGGTC
IL-1β	GCTTCAGGCAGGCAGTATC	AGGATGGGCTCTTCTTCAAAG
IL-6	ACCGCTATGAAGTTCCTCTC	CTCTGTGAAGTCTCCTCTCC
IL-18	ACCAAGTTCTCTTCGTTGAC	TCACAGCCAGTCCTCTTAC
IL-33	CAATGACCAATCTGTTAGT	CATAGTAGCGTAGTAGCA
TNF-α	GGTTCTGTCCCTTTCACTCAC	TGCCTCTTCTGCCAGTTCC
AIM2	ATAGGAGGAACAACAACAT	GCCATCTTCTGCTACATA
ASC	AGGAGTGGAGGGGAAAGC	AGAAGACGCAGGAAGATGG
CASP1	AGGAATTCTGGAGCTTCAATCAG	TGGAAATGTGCCATCTTCTTT
CASP11	GCTCTTACTTCATCACTA	AATATCTCGTCAAGGTTG
NLRP1	GGTGTGCTGGTTGGTCTGC	GTGCTGTGGTGGTCTGTGAG
NLRP3	GCTCCAACCATTCTCTGACC	AAGTAAGGCCGGAATTCACC
NLRP6	GGACGAGAGGAAGGCAGAG	GCACACGAAGGGCACAAAG
NLRP12	AAGAGATGAGATGCTACCTTGAGAG	ATGCCAACACTTCCTCCTTCAC
GFAP	GGTTGAATCGCTGGAGGAG	CTGTGAGGTCTGGCTTGG
Iba-1	TTCCCAAGACCCACCTAG	TCCTCATACATCAGAATCATTC
RPLO	ACTGGTCTAGGACCCGAGAAG	TCCCACCTTGTCTCCAGTCT
RPL27	ACATTGACGATGGCACCTC	GCTTGGCGATCTTCTTCTTG
GAPDH	TGTGTCCGTCGTGGATCTGA	CCTGCTTCACCACCTTCTTGA
β-Actin	AACCGTGAAAAGATGACCCAG	GCCTGGATGGCTACGGCTACGTACATG

## Data Availability

All data associated with the manuscript are included in the manuscript.
